# A hidden epidemic in the concrete jungle: habitat fragmentation and rising snakebite occurrence in São Paulo, a tropical megacity

**DOI:** 10.29374/2527-2179.bjvm002926

**Published:** 2026-09-30

**Authors:** Renan de Oliveira Nogueira, Beatriz Rodrigues da Cruz, Pedro Augusto Pires Brandão, Herbert Sousa Soares, Arlei Marcili, Guilherme José da Costa Silva

**Affiliations:** 1 Programa de Pós-Graduação em Saúde Única, Universidade Santo Amaro (UNISA), São Paulo, SP, Brazil.

**Keywords:** One Health, public health, deforestation, habitat loss, human-snake conflict, human-wildlife conflict, Saúde Única, saúde pública, desmatamento, perda de habitat, conflito humano-serpente, conflito humano-fauna silvestre

## Abstract

This study analyzes 13,111 snakebite cases (2010-2023) in São Paulo, Brazil, correlating them with deforestation patterns using SINAN and MapBiomas data. Snakebite occurrence was concentrated in the southern zone, particularly in the Parelheiros district, an area characterized by intense urban expansion, fragmented forest remnants, and high social vulnerability. *Bothrops* species were responsible for 81.5% of envenomings, with incidents concentrated in three districts bordering fragmented forests. Annual deforestation fluctuated (43.1–321.2 ha), but snakebite patterns showed unique temporal dynamics, including a 2012 outbreak potentially linked to habitat fragmentation from the Rodoanel highway expansion. Intermediate deforestation zones (average of 137.5 ha/year) experienced the highest risks, suggesting edge effects and rodent proliferation as key mediators. The results demonstrate how urban expansion in biodiversity hotspots exacerbates human-wildlife conflict, highlighting the need for integrated prevention strategies in urban-forest interface zones.

## Resumo

Este estudo analisa 13.111 casos de acidentes ofídicos (2010–2023) em São Paulo, Brasil, correlacionando-os com os padrões de desmatamento por meio de dados do SINAN e do MapBiomas. A ocorrência de acidentes ofídicos concentrou-se na zona sul, particularmente no distrito de Parelheiros, uma área caracterizada por intensa expansão urbana, remanescentes florestais fragmentados e elevada vulnerabilidade social. Espécies do gênero *Bothrops* foram responsáveis por 81,5% dos envenenamentos, com os acidentes concentrados em três distritos limítrofes a fragmentos florestais. O desmatamento anual apresentou variação (43,1–321,2 ha), enquanto os acidentes ofídicos demonstraram uma dinâmica temporal particular, incluindo um aumento expressivo em 2012, potencialmente relacionado à fragmentação do habitat decorrente da expansão do Rodoanel. As áreas com níveis intermediários de desmatamento (média de 137,5 ha/ano) apresentaram os maiores riscos, sugerindo que os efeitos de borda e a proliferação de roedores podem atuar como importantes fatores mediadores. Os resultados demonstram como a expansão urbana em áreas de alta biodiversidade intensifica os conflitos entre seres humanos e animais silvestres, destacando a necessidade de estratégias integradas de prevenção nas zonas de interface urbano-florestal.

## Introduction

The Municipality of São Paulo (MSP) is located on the Atlantic Plateau, adjacent to the Serra do Mar mountain range (Almeida, 1964), within the Atlantic Forest biome, one of the world's most threatened ecosystems. Over the past 500 years, this biodiversity hotspot has undergone intense anthropogenic disturbance (Eisenlohr et al., 2015; Morellato & Haddad, 2000; Ribeiro et al., 2011), retaining only 24% of its original forest cover (Fundação SOS Mata Atlântica, 2022). Recognized as a global biodiversity hotspot (Myers et al., 2000), the Atlantic Forest harbors exceptional concentrations of endemic and endangered species (Eisenlohr et al., 2015). The remaining vegetation consists predominantly of small, fragmented forest patches (Vancine et al., 2024). This fragmentation heightens risks for both native wildlife and urban human populations by increasing contact zones, particularly with venomous snakes (Nori et al., 2014; Resiere et al., 2018), and acts as a key driver of disease emergence under One Health principles (Destoumieux-Garzón et al., 2018).

As the largest metropolis in the Southern Hemisphere, São Paulo presents a paradoxical landscape: while 54% of its territory retains vegetation cover, approximately half of this is concentrated in the Parelheiros district alone (Freitas & Pereira, 2017). Although studies document partial vegetation recovery (Amato-Lourenço et al., 2016), regeneration rates show marked spatial disparities reflecting unequal land-use patterns and real estate expansion (Brancalion et al., 2016), a dynamic that may be contributing to the rising occurrence of snakebites in the metropolitan area (Benjamin et al., 2020). Against this backdrop, the present study examines the spatiotemporal relationships between deforestation patterns and snakebite occurrences across São Paulo's administrative regions, with particular focus on identifying high-risk zones where forest loss coincides with elevated envenoming cases. Furthermore, we evaluate how recent urban expansion has altered human-snake conflict dynamics, providing critical data for targeted public health interventions in the rapidly changing ecological matrix of this megacity.

## Materials and methods

This research was designed as a case study focused on statistical analysis of snakebite incidents in the city of São Paulo, using data from public databases and specialized technological platforms. The analysis was conducted on two fronts: (1) snakebite occurrences and (2) vegetation suppression, to evaluate the relationship between habitat fragmentation and snakebite incidence.

Snakebite data were obtained from the TABNET platform, maintained by the Department of Informatics of the Unified Health System (Brasil, 2025). The period analyzed spans from 2010 to 2023, with data from the Notifiable Diseases Information System (SINAN). Data retrieval was performed on July 13, 2024, through the Electronic Citizen Information System (e-SIC), under request protocol number 081545, ensuring data authenticity and reliability. The snakebite data are presented at the municipal level and include the case locations, accident severity, and victim demographic characteristics, enabling a detailed analysis of spatial and temporal patterns.

For the analysis of vegetation cover and forest suppression in the Municipality of São Paulo, we used the MapBiomas platform, which provides land-use and land-cover data and specializes in tracking changes in forest cover over time. The available data span the period from 2010 to 2023, enabling temporal visualization of vegetation cover dynamics. The platform employs satellite imagery and advanced remote sensing techniques to map deforested areas and identify forest habitat fragmentation, with emphasis on the remaining Atlantic Forest and peripheral urban zones. Deforested areas were identified and quantified across the city zones.

## Results

From 2010 to 2023, a total of 13,141 snakebites were reported, although most lacked details regarding the exact location and snake species involved. Among cases with complete records, approximately 81.5% (n = 1,601) came from interactions with snakes of the genus *Bothrops*. Other incidents involved *Crotalus* (3.1%, n = 60), *Micrurus* (1.4%, n = 27), *Lachesis* (0.2%, n = 4), and non-venomous snakes (12.8%, n = 272).

The year 2012 recorded the highest number of snakebite incidents was recorded in 2012, with most cases occurring in the Parelheiros district, accounting for 60% (n = 21) of cases with a known origin. This represents a marked deviation from the period average, during which Parelheiros, although consistently the district with the highest number of incidents, typically accounted for about 23% of known cases. The data further reveal a highly uneven distribution: only ten of the 96 districts reported no incidents. In contrast, the majority of cases were concentrated in just three districts: Parelheiros, Grajaú, and Jardim Ângela.

Deforestation rates in the city of São Paulo indicate that between 2010 and 2023, a total of 321.000 hectares were cleared. Over this period, deforestation rates fluctuated considerably. The highest deforestation rate (321.2 hectares) was recorded in 2015, and the lowest (43.1 hectares) was in 2023. The annual average was 137.5 hectares. In general, deforestation rates declined in the final years of the series, except for 2021, which recorded the second-highest value (231.1 hectares).

## Discussion

Since the dawn of civilization, snakebite accidents have been a significant public health concern. These incidents can result in minor local injuries, limb loss, or even death. Tropical countries like Brazil experience a higher incidence of cases due to snakes’ adaptability to both rural and urban environments (Araújo et al., 2003; Baldassin et al., 2021; Mise et al., 2007; Moreno et al., 2005). According to the World Health Organization (2019), snakebites cause an estimated 81,000 to 138,000 deaths annually worldwide, underscoring the severity of this issue.

Snakebite envenoming remains a highly neglected global health issue, particularly in socioeconomically vulnerable regions (Ceríaco & Marques, 2021). This neglect prompted the WHO to classify snakebite envenoming as a Category A Neglected Tropical Disease, the most severe designation. In São Paulo, only 15% of snakebite cases are fully documented, including information on the species involved, and only 78% include the geographic location. This lack of data hampers effective treatment and hinders prevention efforts in high-risk communities (Gutiérrez et al., 2017).

The Brazilian Ministry of Health provides free antivenom to the population; however, insufficient data collection continues to undermine effective monitoring and control of accidents (Schneider et al., 2021). Alarmingly, this issue has persisted virtually unchanged for over a century (Bochner & Struchiner, 2003). Harrison et al. (2009) highlight that poor record-keeping in snakebite cases is a global problem, particularly in low-income regions, further exacerbating public health challenges. In the same study, the authors further emphasize that snakebite is a health issue closely linked to poverty, justifying its classification as a Neglected Tropical Disease. Therefore, any epidemiological association must be interpreted in light of the broader socioeconomic landscape alongside environmental factors. In São Paulo, this dynamic is starkly evident. As Figure 1 shows, the districts with the highest numbers of snakebites coincide, in most cases, with those presenting the highest social vulnerability indices. Parelheiros, specifically, presents an average IPVS score of 4.3 (indicating high social vulnerability) and, concomitantly, the highest incidence of snakebites during the study period. At the same time, the locations with the largest deforestation hotspots are also the districts with the highest bite rates, as shown in the same figure. In other words, poverty alone cannot be interpreted as an isolated risk factor; rather, the pattern of occupation in forested peripheries by marginalized populations exposes them to a greater risk of snakebite incidents. Thus, the high incidence in this region is not merely an ecological consequence of living near forest remnants. It is also a direct reflection of the convergence between extreme urban poverty, informal expansion, and environmental degradation (Alves et al., 2020; Ceríaco & Marques, 2021).

**Figure 1:**
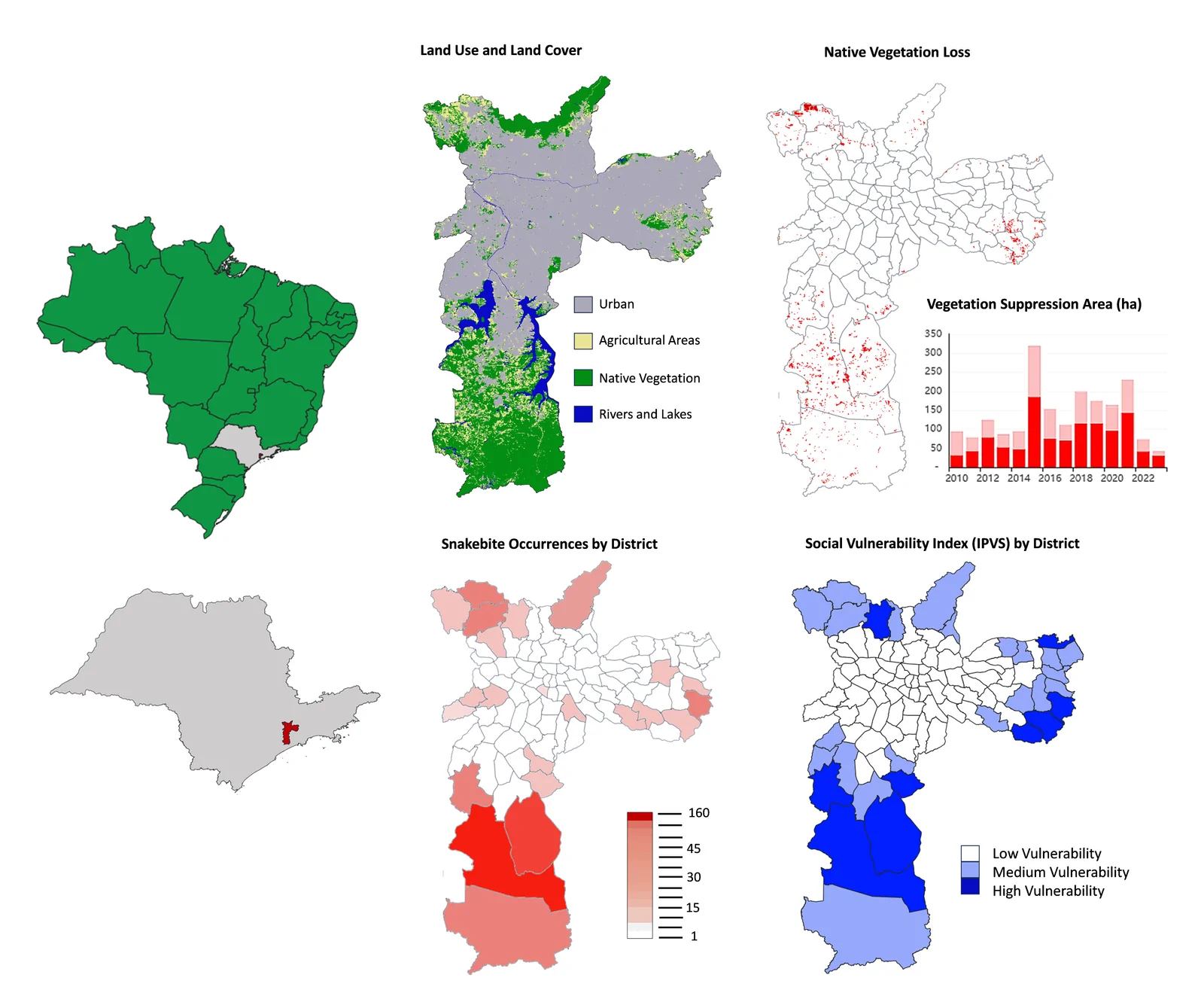
Spatial, environmental, and socioeconomic analysis of snakebite incidents in the municipality of São Paulo (2010–2023). The composition includes contextual maps of the municipality's geographic location within Brazil, along with detailed maps depicting Land Use and Land Cover, Native Vegetation Loss, Snakebite Occurrences by District, and the Social Vulnerability Index (IPVS) by District. Additionally, the figure features a bar chart detailing the annual Vegetation Suppression Area (ha) and a dual-axis line graph illustrating the temporal trends of Deforestation (ha) versus Recorded snakebites over the study period.

To fully grasp this convergence, the ecological context of these marginalized peripheries must be highlighted. Located within the Atlantic Forest biome, it is home to medically significant snakes, including *Bothrops* (lanceheads), *Crotalus* (rattlesnakes), and *Micrurus* (coral snakes) (Marques et al., 2009). Approximately 18% of the city’s territory remains forested, primarily in the southern zone (Gaudereto et al., 2017) (Figure 1). This southern region of São Paulo recorded the highest loss of vegetation cover between 2010 and 2019, with deforestation driven mainly by irregular settlements and pasture expansion. This region also reported the highest number of snakebites in the city. The Parelheiros district had the highest incidence of snakebites and the most significant deforestation during the study period. Snakebites are more frequent in rural communities near forest edges and tend to peak during warmer months. A study by Nogueira et al. (2019) confirms that habitat destruction increases human-snake conflicts, particularly in tropical regions.

In 2012, the Parelheiros district recorded an unusually high number of snakebites, accounting for 60% of the city’s total cases. Although a slight increase in deforestation was observed (Figure 2), no direct correlation was established because some years, such as 2015, experienced greater deforestation without a proportional rise in snakebite incidents. The 2012 spike may be linked to habitat fragmentation and increased human encroachment into forest remnants (Myers et al., 2000; Varjabedian, 2010). Notably, the southern section of the Rodoanel Mário Covas highway opened two years earlier, accelerating regional occupation (Queiroz et al., 2014).

**Figure 2:**
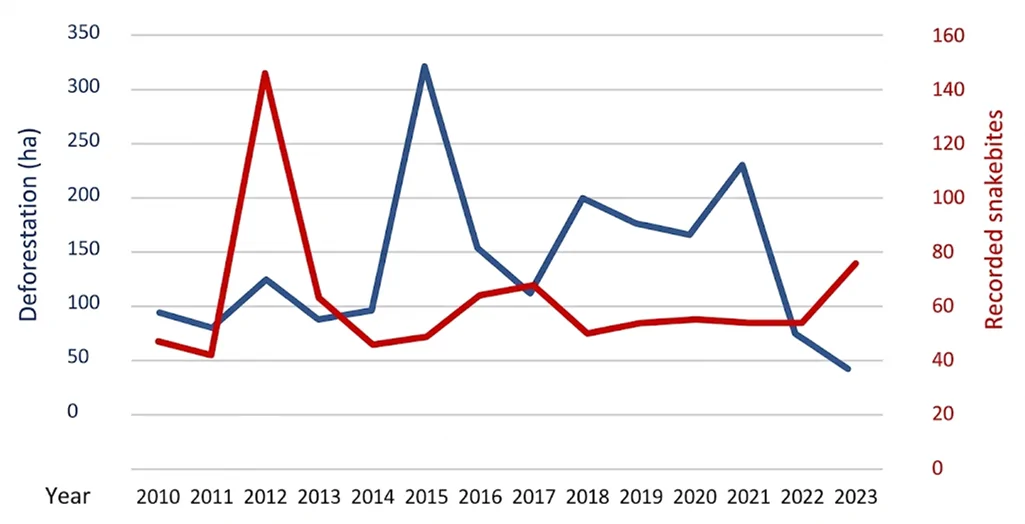
Deforestation trends (blue) and snakebite incidents (red) in the city of São Paulo from 2010 to 2023. Data sources: Brazilian Ministry of Health/Health Surveillance Secretariat (SVS) - Notifiable Diseases Information System (SINAN Net) and MapBiomas (Generated by authors).

Thus, irregular settlements in forested areas are high-risk zones for snakebites both during and after vegetation clearance. Additionally, waste dumping promotes rodent proliferation, the primary prey of adult lancehead vipers, and discarded materials may serve as shelters for snakes. A study by Alves et al. (2020) emphasizes that poor urban planning in peri-forest zones significantly increases the risk of snakebites.

## Conclusion

Snakebite envenoming remains a critical yet underrecognized public health challenge, particularly in tropical regions such as Brazil. The lack of comprehensive epidemiological data, coupled with habitat destruction and urban expansion, exacerbates the risks faced by vulnerable communities. While antivenom distribution is a crucial component of care, effective prevention requires enhanced surveillance, community education, and the implementation of sustainable land-use policies. Addressing these gaps demands interdisciplinary collaboration, integrating ecology, public health, and urban planning to mitigate human-snake conflicts. As emphasized by the World Health Organization (2023), reducing snakebite-related mortality and disability by 2030 requires urgent action, beginning with improved data collection and targeted interventions in high-risk areas such as São Paulo’s southern zone. 

## References

[B001] Almeida F. F. M. (1964). Fundamentos geológicos do relevo paulista.

[B002] Alves R. R. N., Silva V. N., Trovão D. M. B. M., Oliveira J. V., Mourão J. S., Dias T. L., Alves Â. G. C., Lucena R. F. P., Barboza R. R. D., Souto W. M. S. (2020). Factors associated with snakebite risk in tropical forest regions: A systematic review. Journal of Ethnobiology and Ethnomedicine.

[B003] Amato-Lourenço L. F., Moreira T. C. L., Arantes B. L. D., Silva D. F. D., Mauad T. (2016). Metrópoles, cobertura vegetal, áreas verdes e saúde. Estudos Avançados.

[B004] Araújo F. A. A., Santalúcia M., Cabral R. F., Cardoso J. L. C., Haddad V., França F. S., Malaque C. M. S., Wen W. (2003). Animais peçonhentos no Brasil: Biologia, clínica e terapêutica dos acidentes.

[B005] Baldassin J. C. S., Francisco S. R., Silva R. W., Moura R. F., Pombo A. P. M. M. (2021). Perfil epidemiológico e dinâmica da distribuição dos acidentes ofídicos em humanos no Estado de São Paulo. Hygeia; Revista Brasileira de Geografia Médica e da Saúde.

[B006] Benjamin J. M., Abo B. N., Brandehoff N. (2020). Snake envenomation in Africa. Current Tropical Medicine Reports.

[B007] Bochner R., Struchiner C. J. (2003). Epidemiologia dos acidentes ofídicos nos últimos 100 anos no Brasil: Uma revisão. Cadernos de Saúde Pública.

[B008] Brancalion P. H., Garcia L. C., Loyola R., Rodrigues R. R., Pillar V. D., Lewinsohn T. M. (2016). A critical analysis of the Native Vegetation Protection Law of Brazil (2012): Updates and ongoing initiatives. Natureza & Conservação.

[B009] Brasil (2025). Acidente por animais peçonhentos - Notificações registradas no Sistema de Informação de Agravos de Notificação (SINAN).

[B010] Ceríaco L. M., Marques M. P. (2021). Serpentes venenosas e acidentes ofídicos. Revista de Ciência Elementar.

[B011] Destoumieux-Garzón D., Mavingui P., Boetsch G., Boissier J., Darriet F., Duboz P., Fritsch C., Giraudoux P., Le Roux F., Morand S., Paillard C., Pontier D., Sueur C., Voituron Y. (2018). The One Health Concept: 10 years old and a long road ahead. Frontiers in Veterinary Science.

[B012] Eisenlohr P. V., Oliveira-Filho A. T., Prado J. (2015). The Brazilian Atlantic Forest: New findings, challenges and prospects in a shrinking hotspot. Biodiversity and Conservation.

[B013] Freitas J. C., Pereira A. C. (2017). Ocupações irregulares em São Paulo. Revista de Pós-graduação Multidisciplinar.

[B014] Fundação SOS Mata Atlântica (2022). Atlas dos remanescentes florestais da Mata Atlântica – Período 2020-2021.

[B015] Gaudereto G. L., Aguiar A. O., Gallardo A. L. C. F. (2017). Plano Municipal da Mata Atlântica de São Paulo: Oficinas participativas como parte do processo de planejamento. Revista de Gestão Ambiental e Sustentabilidade.

[B016] Gutiérrez J. M., Calvete J. J., Habib A. G., Harrison R. A., Williams D. J., Warrell D. A. (2017). Snakebite envenoming. Nature Reviews. Disease Primers.

[B017] Harrison R. A., Hargreaves A., Wagstaff S. C., Faragher B., Lalloo D. G. (2009). Snake envenoming: A disease of poverty. PLoS Neglected Tropical Diseases.

[B018] Marques O. A. V., Pereira D. N., Barbo F. E., Germano V. J., Sawaya R. J. (2009). Os répteis do município de São Paulo: Diversidade e ecologia da fauna pretérita e atual. Biota Neotropica.

[B019] Mise Y. F., Lira-da-Silva R. M., Carvalho F. M. (2007). Envenenamento por serpentes do gênero Bothrops no Estado da Bahia: Aspectos epidemiológicos e clínicos. Revista da Sociedade Brasileira de Medicina Tropical.

[B020] Morellato L. P. C., Haddad C. F. B. (2000). Introduction: The Brazilian Atlantic Forest. Biotropica.

[B021] Moreno E., Queiroz-Andrade M., Lira-da-Silva R. M., Tavares-Neto J. (2005). Características clínico-epidemiológicas dos acidentes ofídicos em Rio Branco, Acre. Revista da Sociedade Brasileira de Medicina Tropical.

[B022] Myers N., Mittermeier R. A., Mittermeier C. G., Fonseca G. A. B., Kent J. (2000). Biodiversity hotspots for conservation priorities. Nature.

[B023] Nogueira C. C., Argôlo A. J. S., Arzamendia V., Azevedo J. A., Barbo F. E., Bérnils R. S., Borges-Martins M., Brandão R. A., Braz H. B., Buononato M. A., Cisneros-Heredia D. F., Colli G. R., Costa H. C., Franco F. L., Giraudo A., Gonzalez R. C., Guedes T. B., Hoogmoed M. S., Marques O. A. V., Martins M., Mônico A. T., Passos P., Prudente A. L. C., Rivas G. A., Scrocchi G. J., Silva N. J., Strüssmann C., Vieira-Alencar J. P. S., Zaher H. (2019). Atlas of Brazilian snakes: Verified point-locality maps to mitigate the Wallacean shortfall in a megadiverse snake fauna. South American Journal of Herpetology.

[B024] Nori J., Carrasco P. A., Leynaud G. C. (2014). Cobras venenosas e mudanças climáticas: Ofidismo como um problema dinâmico. Climatic Change.

[B025] Queiroz F. T., Aguiar A. O., Brito S. C., Bueno H. G. (2014). Gestão ambiental em obras viárias: Estrutura e resultados no caso do Rodoanel Mário Covas, trecho sul. Revista Metropolitana de Sustentabilidade.

[B026] Resiere D., Mehdaoui H., Gutiérrez J. M. (2018). Snakebite envenomation in the Caribbean: The role of medical and scientific cooperation. PLoS Neglected Tropical Diseases.

[B027] Ribeiro M. C., Martensen A. C., Metzger J. P., Tabarelli M., Scarano F., Fortin M. J., Zachos F. E., Habel J. C. (2011). Biodiversity hotspots: Distribution and protection of conservation priority areas.

[B028] Schneider M. C., Min K. D., Hamrick P. N., Montebello L. R., Ranieri T. M., Mardini L., Camara V. M., Raggio Luiz R., Liese B., Vuckovic M., Moraes M. O., Lima N. T. (2021). Overview of snakebite in Brazil: Possible drivers and a tool for risk mapping. PLoS Neglected Tropical Diseases.

[B029] Vancine M. H., Muylaert R. L., Niebuhr B. B., Oshima J. E. F., Tonetti V., Bernardo R., De Angelo C., Rosa M. R., Grohmann C. H., Ribeiro M. C. (2024). The Atlantic Forest of South America: Spatiotemporal dynamics of the vegetation and implications for conservation. Biological Conservation.

[B030] Varjabedian R. (2010). Lei da Mata Atlântica: Retrocesso ambiental. Estudos Avançados.

[B031] World Health Organization (2019). Snakebite envenoming: A strategy for prevention and control..

[B032] World Health Organization (2023). Snakebite envenoming.

